# Integration of Through‐Sapphire Substrate Machining with Superconducting Quantum Processors

**DOI:** 10.1002/adma.202411780

**Published:** 2025-01-19

**Authors:** Narendra Acharya, Robert Armstrong, Yashwanth Balaji, Kevin G. Crawford, James C. Gates, Paul C. Gow, Oscar W. Kennedy, Renuka Devi Pothuraju, Kowsar Shahbazi, Connor D. Shelly

**Affiliations:** ^1^ Oxford Quantum Circuits Thames Valley Science Park Shinfield, Reading RG2 9LH UK; ^2^ Optoelectronics Research Centre University of Southampton Southampton SO17 1BJ UK

**Keywords:** dielectric, Josephson junctions, machining, nanofabrication, qubits

## Abstract

A sapphire machining process integrated with intermediate‐scale quantum processors is demonstrated. The process allows through‐substrate electrical connections, necessary for low‐frequency mode‐mitigation, as well as signal‐routing, which are vital as quantum computers scale in qubit number, and thus dimension. High‐coherence qubits are required to build fault‐tolerant quantum computers and so material choices are an important consideration when developing a qubit technology platform. Sapphire, as a low‐loss dielectric substrate, has shown to support high‐coherence qubits. In addition, recent advances in material choices such as tantalum and titanium‐nitride, both deposited on a sapphire substrate, have demonstrated qubit lifetimes exceeding 0.3 ms. However, the lack of any process equivalent of deep‐silicon etching to create through‐substrate‐vias in sapphire, or to inductively shunt large dies, has limited sapphire to small‐scale processors, or necessitates the use of chiplet architecture. Here, a sapphire machining process that is compatible with high‐coherence qubits is presented. This technique immediately provides a means to scale quantum processing units (QPUs) with integrated mode‐mitigation, and provides a route toward the development of through‐sapphire‐vias, both of which allow the advantages of sapphire to be leveraged as well as facilitating the use of sapphire‐compatible materials for large‐scale QPUs.

## Graphical abstract

Through‐sapphire machining is integrated with superconducting quantum processors (QPUs) and qubits. High coherence of the qubits is demonstrated as well as low parameter spread. This machining integration provides a route to further scaling of QPUs on new materials such as tantalum that are typically deposited on sapphire substrates. In addition, the machining provides a route to through‐sapphire‐vias in superconducting circuits.

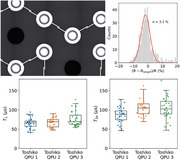

## 1 Introduction

To progress toward fault‐tolerant quantum computing large numbers of qubits are required. Superconducting qubits present a promising platform with which to scale quantum processors.^[^ ^1^, ^2^, ^3^ ^]^ As processor qubit number increases, typically the dimensions of the processors also increase. With this scaling, the size of the quantum processing unit (QPU) can become physically large enough that the dimensions of the cavity or enclosure which houses the processor can support modes that are commensurate with the qubit frequencies. Multiple approaches to avoid these spurious modes have been implemented: on typical planar devices separated ground planes can be inductively shunted with airbridges,^[^ ^4^ ^]^ or connected with through‐substrate‐vias (TSVs).^[^ ^5^, ^6^, ^7^ ^]^ Low‐frequency cavity modes can be avoided by ensuring that the cavities are small enough such that these modes cannot be supported. To this end, quantum processors have been divided into chiplets with each chiplet housed in smaller (≈1 cm) cavities. The processors can also be housed in inductively shunted cavities.^[^ ^8^, ^9^ ^]^ Spring et al. demonstrated an architecture that provides this inductive shunting by means of a conducting pillar passing through an aperture in the substrate and connecting the top and bottom walls of the enclosure.^[^ ^10^ ^]^ TSVs and inductively shunted cavities both require electrical connections to pass through the substrate of the QPU, a critical capability to scale the size of QPUs.

Despite the technological importance of superconducting qubits, they rely on a small number of critical materials. With few exceptions, high‐performance qubits have superconducting pads made from aluminum (Al),^[^ ^1^, ^11^, ^12^ ^]^ niobium (Nb),^[^ ^13^ ^]^ tantalum (Ta),^[^ ^14^, ^15^ ^]^ and titanium‐nitride (TiN)^[^ ^16^ ^]^ or nitrides of the previously listed metals.^[^ ^17^ ^]^ The Josephson junctions (JJs) are typically made from Al/AlOx/Al tunnel barriers and they are manufactured on high resistivity silicon or sapphire substrates. Although both substrates are currently compatible with state‐of‐the‐art performance, sapphire is incompatible with large‐scale integration as there are no processes that allow the through‐substrate electrical connections required for mode mitigation. Historically, this has left just one viable substrate material, silicon, which has been used to integrate many complex 3D layered devices.^[^ ^5^, ^6^, ^18^ ^]^

High‐coherence qubits have been manufactured on silicon substrates; records include *T*_1_ ∼ 300 µs using capped Nb films,^[^ ^13^ ^]^ *T*_1_ ∼ 200 µs using uncapped Nb films^[^ ^19^ ^]^ and *T*_1_ ∼ 270 µs using Al films.^[^ ^11^ ^]^ Whereas on sapphire substrates records include *T*_1_ = 300 − 400 µs using TiN films^[^ ^16^ ^]^ and *T*_1_ = 300 − 400 µs using Ta films.^[^ ^14^, ^15^ ^]^ In the case of tantalum, high‐coherence qubits have only been shown on sapphire. Dielectric loss measurements of sapphire performed at mK show record loss tangents with crystals grown by the heat‐exchanger method (HEM) reporting tanδ_bulk_ = 1.9 × 10^−8^.^[^ ^20^ ^]^ High quality silicon is also likely a very low‐loss substrate as shown by the coherence values referred to above.^[^ ^11^, ^13^, ^19^ ^]^ To the authors best knowledge the lowest reported values of the bulk dielectric constant being tanδ_bulk_ = 2.7 × 10^−6^^[^ ^21^ ^]^ however more investigation of this platform will likely revise this number downward. Which substrate will eventually become the substrate of choice is an open question and may be driven by loss mechanisms yet to be determined. For instance, acceptor losses in silicon may provide a hard‐to‐engineer loss mechanism.^[^ ^22^ ^]^

As sapphire offers a low‐loss platform for high‐coherence qubits, a route toward scaling and mitigating the modes that come with increased chip dimension is required. In this work, we demonstrate a complete end‐to‐end manufacturing process of an Oxford Quantum Circuits (OQC) 32‐qubit QPU “Toshiko” integrated with through‐sapphire machining to incorporate through‐sapphire pillars which inductively shunt the QPU enclosure for mode‐mitigation purposes. The demonstration of high‐coherence qubits on a sapphire substrate that has undergone a computer numerical control (CNC) machining process effectively unlocks sapphire as a technologically relevant platform to scale superconducting qubits.

## 2 Results

### 2.1 Qubit Fabrication and Sapphire Machining Integration

**Figure 1:**
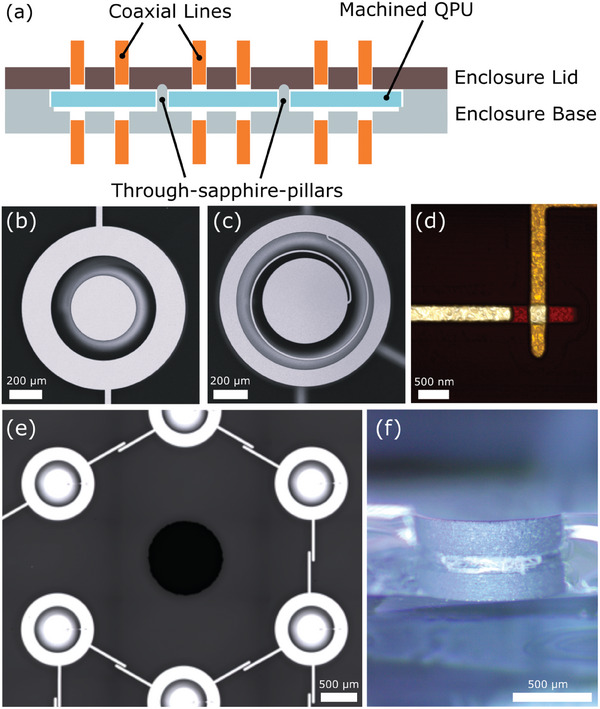
a) Schematic of sapphire QPU with through‐substrate machining. Pillars extrude from enclosure base protruding through the sapphire QPU and make electrical contact with the enclosure lid. Constituent components of a substrate‐drilled coaxmon‐based QPU: b) The qubit electrodes are fabricated on the top side of the wafer using a combination of e‐beam deposition, photolithography and etching processes. c) The lumped LC spiral resonator is similarly fabricated on the back side of the wafer. d) An atomic force microscope (AFM) image of the Dolan bridge Josephson junction. The Josephson junction is defined by electron‐beam lithography and fabricated using a typical double‐angle shadow evaporation technique on the top side of the wafer forming the qubit cell. e) Shows a micrograph of a drilled aperture in sapphire in close vicinity to the qubit lattice. The drilling is performed after all of the fabrication is complete, but prior to wafer dicing. The qubit and resonator outer diameters are 1 mm. f) Shows a profile micrograph of a cleaved sapphire die post‐machining (cleaved through the middle of the aperture). The ridge in the middle of the aperture is due to tool wear. The diameter of the aperture is ≈1 mm. The thickness of the sapphire is ≈500 µm.

The qubits used in this work are coaxmons ‐ an architecture that necessitates fabricating on both sides of a wafer. The coaxmon is an implementation of the transmon whereby the qubit has a coaxial geometry and is fabricated on one side of a substrate while a corresponding readout resonator is a lumped LC spiral resonator and is fabricated on the other side of the substrate aligned to the qubit.^[^ ^23^ ^]^ Each qubit cell is capacitively coupled to control and readout ports, and may be scaled to large 2D qubit arrays. See **Figure** **1** for examples of each constituent component of the coaxmon.

Integration of TSVs in silicon is usually achieved using high aspect ratio chemical etch process such as deep reactive ion etching (DRIE).^[^ ^24^ ^]^ As sapphire is inert to most physical and chemical etching, the high aspect ratio etching processes used ubiquitously for silicon TSVs do not exist for sapphire.^[^ ^25^ ^]^ However, it is possible to create apertures in sapphire using a laser beam in a process known as laser drilling.^[^ ^26^ ^]^ The material removal during laser drilling is an ablative process with a large amount of energy deposited into the substrate which heats the area around the drilling site.^[^ ^27^ ^]^ Due to the extent of this heating we have found laser drilling to be difficult to integrate with our manufacturing process. For more details see Supplemental Material III.

An alternative to etching and laser drilling to create apertures or vias in silicon is CNC machining. Apertures in silicon achieved using CNC machining has been demonstrated on 4‐qubit devices,^[^ ^10^ ^]^ with qubit coherence times in excess of 100 µs.

Despite sapphire's high hardness and relatively low fracture toughness which makes it challenging to process via physical machining, we successfully demonstrate the use of CNC machining as an alternative to plasma etching processes and laser drilling to create through‐sapphire apertures. The apertures were CNC machined in the sapphire using a Loxham Precision µ6 micro‐milling system. A diamond micro‐grain tool measuring 600 µm in diameter, was used to form 1 mm diameter apertures following a helical toolpath. The 600 µm tool size was chosen to allow clearance for evacuation of debris during machining. The spindle speed used was 125 000 rpm. During drilling, the substrate and grinding pin are actively cooled using deionised (DI) water (≈0.1 l min^−1^ of DI water); this keeps the grinding pin cool, but also mitigates the heating of the Josephson junctions and protective coatings. The substrate is coated in a series of charge mitigation and photoresist layers to protect the nanofabricated circuits from electrostatic damage as well as machining debris. The drilling takes ≈3 mins per hole. Note that heat affecting the JJs can be avoided by machining the apertures in advance of fabricating the JJs. However, the resulting resist inhomogeneity due to apertures being present during resist‐spinning leads to devices with increased wafer‐scale resistance spread. This is in‐line with the results of Muthusubramanian et al.^[^ ^28^ ^]^ where they performed a study of both Dolan Bridge and Manhattan style Josephson junctions with and without integrated TSVs. They showed that in both styles of JJs the wafer‐scale resistance spread is increased on a TSV‐integrated substrate and is attributed to an increase in resist‐height variation on the TSV‐integrated substrates when compared to planar substrates.

### 2.2 Josephson Junction Sensitivity to Machining

**Figure 2:**
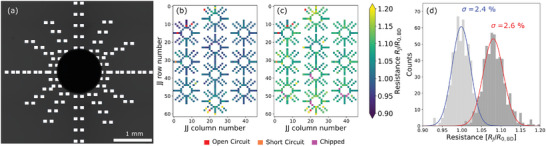
The figure demonstrates the robustness of the Josephson junctions to the mechanical drilling process. a) A micrograph of a radial test pattern of Josephson junctions. A 1 mm aperture was machined through the sapphire in close vicinity to the junctions. The aperture is surrounded by 56 JJs in a radial pattern. b) A composite resistance color‐map of 10 drilled apertures before machining. The JJ resistances are measured prior to drilling and any failures such as a short‐circuit or an open‐circuit are recorded (shown as orange and red on the colormap respectively). c) JJ resistance measurements of the same die after aperture machining. Any additional JJ failures are recorded. In addition, we record JJs that were unmeasurable due to chipping of the sapphire. d) Histograms of the JJ resistances prior to, and after, the sapphire machining process. Prior to machining the resistance spread is σ = 2.4 %. After the machining, the resistance spread is slightly increased to σ = 2.6 %. Note that there is also a shift of the die median after machining. This shift in resistance may be due to the time between subsequent measurements (known as aging), as a result of the machining itself, or a combination of both effects.

To determine the robustness of the Josephson junctions to the drilling process, a test wafer was fabricated with JJs placed in a radial pattern surrounding where the substrate machining would be performed. The pattern was made to determine if there was a higher incidence of failures close to the drilling site, as well as to determine if the machining processes causes any residual heating that can affect the junctions due to heat‐induced accelerated aging.^[^ ^29^ ^]^ **Figure** **2**a shows the radial JJ test dies with an ≈1 mm diameter aperture machined in the center. Figure 2b,c shows the resistance color‐maps before and after the substrate machining process, respectively. Failure mechanisms such as open‐circuits and short‐circuits are recorded. Damage from sapphire chipping is also recorded in the post‐machined map (c). The histograms in Figure 2d confirm that the increase in JJ resistance spread after the drilling process is small (σ = 2.4 % before drilling and σ = 2.6 % after drilling). We see no clear spatial dependence of the JJs resistance change in relation to the aperture machining site. The fabrication JJ yield (JJs not short‐circuit or open‐circuit) of the devices shown in Figure 2b was 98.9 %. After the drilling 14 JJs could not be measured due to chipping of the pads very close to the drill location. No additional short‐circuit or open‐circuits were caused by the drilling in this die. (We note that in a co‐drilled die an additional 4 short‐circuits were measured post‐drilling, representing a 99.3 % drilling success rate).

### 2.3 Full Wafer QPU Production and Coherence Measurements

**Figure 3:**
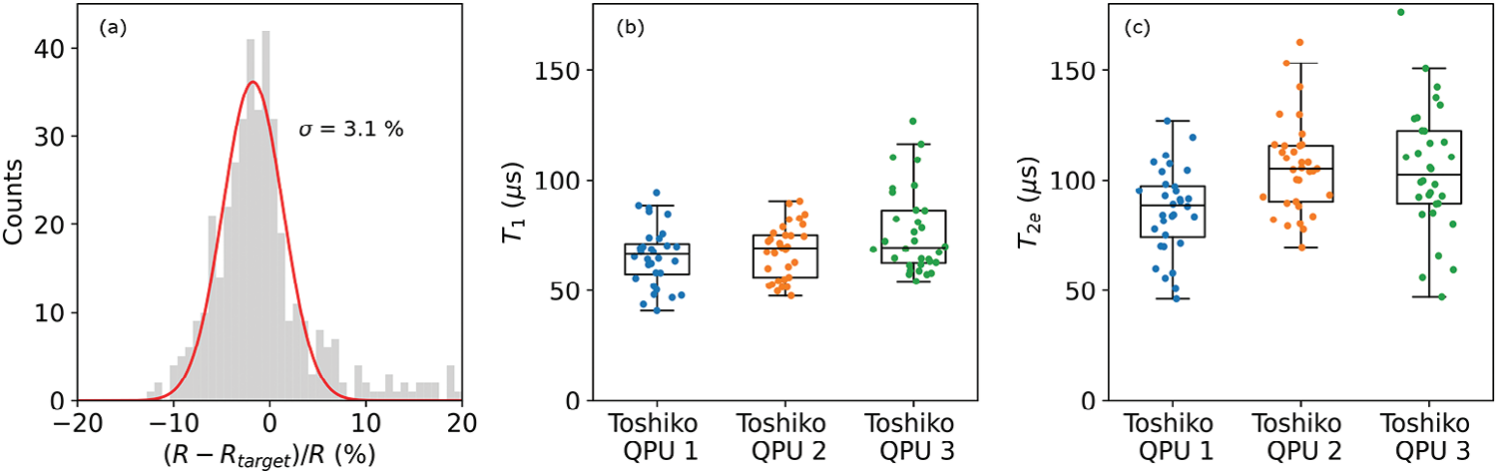
a) Histogram of Josephson junction resistances measured on a 3 ″ wafer of Toshiko 32Q QPUs. The histogram is a composite of three sets of JJs normalized to their median, or target value *R*_target_, showing spread in resistance of 3.1 %. The resistances are measured at the end of the full fabrication process inclusive of both sapphire aperture machining, and dicing; thus the spread represents the full end‐to‐end QPU process. b) Boxplots of 3 × 32Q QPU *T*_1_ relaxation times. Each circle is a median value for 50+ measurements. c) Boxplots of 3 × 32Q QPU *T*_2*e*_ coherence times. Each circle is a median value for 50+ measurements. characteristic decay times. The full‐QPU median values are shown in Table 1.

For our 32Q QPU manufacturing, we use 3 ″ double‐sided‐polished sapphire as the substrate. The aperture machining process is performed after fabrication. The wafer is then diced into individual QPUs prior to final cleaning. The electrical resistance of the Josephson junctions are measured using a probe station to ascertain if any of the JJs have become damaged (for instance a short‐circuit or open‐circuit). **Figure** **3**a shows a histogram of resistance values measured on a representative 3 ″ wafer of Toshiko 32Q QPUs with a spread of resistance values σ = 3.1 %. Resistance spread is computed by first grouping qubits by frequency target, finding the median resistance value in each group and computing the percentage deviation of the resistance of the junction in each qubit from the median of its group. We then create a histogram of the resulting percentage distribution with 50 bins from –20 to +20 % and fit a normal distribution to the histogram, reporting the standard deviation of the normal distribution. Note that the resistances presented are measured at the end of the full manufacturing process, inclusive of fabrication, sapphire aperture machining, and dicing. This demonstrates that our process is able to produce wafer‐scale Josephson junction spread commensurate with state‐of‐the‐art values whilst incorporating additional machining of the QPU. Recent improvements in our Josephson junction fabrication have reduced this spread to 2.4 % at 3 ’’ wafer‐scale (see Section SI, Supporting Information) without any JJ post‐processing such as laser‐tuning,^[^ ^30^ ^]^ electron‐beam‐tuning,^[^ ^31^ ^]^ or voltage‐tuning.^[^ ^32^ ^]^

To demonstrate that the sapphire machining process is compatible with the manufacture of high‐coherence qubits and QPUs, measurements of *T*_1_ and *T*_2*e*_ coherence statistics for three OQC Toshiko 32Q QPUs are presented. The Toshiko 32Q QPU has eight 1 mm apertures machined through the sapphire substrate allowing conducting pillars to pass through the substrate to connect the top and bottom of the QPU cavity. This forms the inductive shunt which allows the formation of architectures that enable cavity mode mitigation as discussed in Section 1.^[^ ^9^, ^10^ ^]^

The *T*_1_ relaxation time and *T*_2*e*_ coherence times statistics are presented for the Toshiko 32Q QPUs as shown in Figure 3b,c respectively. As per coherence‐reporting best‐practice, median values of a statistically significant number of coherence measurements are reported.^[^ ^33^ ^]^ Each data point in the plot represents a single qubit and the value plotted is a median of 50+ decay time measurements. The full‐QPU median values are presented in **Table** **1**.

**Table 1:** Table presents the *T*_1_ and *T*_2*e*_ coherence data from 3 Toshiko QPUs. Full‐QPU median values are shown for each characteristic decay time.

QPU	*T*_1_[µs]	*T*_2*e*_[µs]
Toshiko‐1	66.4 ± 13.5	88.6 ± 19.5
Toshiko‐2	69.2 ± 12.0	105.2 ± 21.3
Toshiko‐3	69.4 ± 19.5	102.6 ± 28.6

The single‐qubit‐median best values are *T*_1_ = 127 µs and *T*_2*e*_ = 176 µs. Also notable are the single‐qubit‐median worst values where *T*_1_ = 40 µs and *T*_2*e*_ = 42 µs representing 60%, and 47%, of the ensemble median values of a total of 96 qubits. The absence of qubits with anomalously low coherence is highly desirable for QPUs, as it means that algorithmic chains are not broken by error hot‐spots. The coherence times reported here are in‐line with those on silicon substrates in pillar‐integrated packages at smaller scale as demonstrated in Spring et al.,^[^ ^10^ ^]^ as well with uncoupled qubit coherence values from OQC 8Q devices as shown in Ref. [31]. The coherence times are an improvement over OQC's previous generation Lucy 8Q QPU^[^ ^34^ ^]^ despite scaling in qubit count, die size, and integrating the additional sapphire machining process steps. As the coherence numbers reported in Figure 3 are from a Toshiko‐generation quantum computer, coupling rates are targeted for aggregate performance i.e. gate and readout fidelities. It is likely that by reducing coupling rates these coherence times could be further improved, showing the ultimate material‐platform limit of our end‐to‐end process and the numbers presented are a lower bound on this. We can place limits on drilling‐related losses by comparing the coherence results presented in Figure 3 to those presented in Ref. [31] for an un‐drilled coaxmon chip made with a similar material platform which showed median *T*_1_ of ≈100 µs. Assuming that any reduction in *T*_1_ is from material damage caused by drilling, we infer an upper bound on drilling‐related loss of 1/212 µs^−1^, however the presence of qubits with *T*_1_ times substantially above the median in Figure 3 indicates that it is unlikely that it is as high as this. Our material platform is likely limited by materials surfaces, metal quality, and buried oxides formed during fabrication. Addressing each of these loss mechanisms is compatible with our drilling process.

## 3 Discussion

We have demonstrated a technique to create through‐sapphire apertures and have integrated this with a 32‐qubit QPU. We demonstrate high yield of the Josephson junctions following the full fabrication flow and the post‐fabrication drilling and dicing. We have shown by measurements of *T*_1_ and *T*_2*e*_ coherence statistics for our Toshiko 32Q QPU processor that the mechanical drilling technique is compatible with a high‐coherence qubit platform. This work creates an opportunity for the quantum computing field to further explore promising low‐loss dielectric substrates such as sapphire. As such, it encourages further development of material platforms such as tantalum (deposited on sapphire) as they now can also be scaled further. Although we demonstrate the through‐sapphire apertures for the purpose of inductive shunt package integration, the results are promising for future development of through‐sapphire‐vias, specifically for the purpose of signal routing and delivery. We demonstrate proof‐of‐principle vias using electron‐beam evaporation in Section S2 (Supporting Information). This work also unlocks the potential for sapphire as a low‐loss dielectric for QPU‐adjacent quantum interposers.

## 4 Experimental Section

### Fabrication

The qubits were fabricated on 3’’ double‐sided‐polished sapphire. Aluminium was deposited on one side of the wafer and then qubit electrodes were formed using photolithography and etching. The process was repeated on the back side of the wafer to form the coaxial resonators. Finally the Josephson junctions were fabricated using a typical Dolan Bridge double‐angle shadow evaporation deposition.^[^ ^35^ ^]^ The QPUs were then machined using the Loxham CNC before being diced into individual QPU dies.

## Conflict of Interest

We have submitted a patent filing on part of the fabrication flow.

## Author Contributions

K.S., N.A., Y.B., R.F.A., K.G.C., and R.D.P. fabricated the devices and QPUs, carried out the JJ resistance measurements, and performed the microscopy. PCG and JCG developed the sapphire machining process. KS, NA, YB, RFA, KGC, RDP, OWK, and CDS developed the process flow to integrate the aperture machining with QPUs and JJs. PCG carried out the aperture machining. YB and CDS oversaw machining of the QPUs and JJs. KS, OWK, and CDS performed the analysis on the data. KS developed and fabricated the aperture metalisation via proof‐of‐prinicple. CDS wrote the manuscript with significant input from KS and OWK. CDS led the project.

## Supporting information

Supporting Information

## Data Availability

The data that support the findings of this study are available from the corresponding author upon reasonable request.
