# Supplementary material for: Integration of Through‐Sapphire Substrate Machining with Superconducting Quantum Processors
Source: Adv Mater. 2025 Jan 19;37(9):2411780. doi: 10.1002/adma.202411780 (PMC11881675; doi:10.1002/adma.202411780)
Supplement: Supplementary file 1 — Supporting Information [file ADMA-37-2411780-s001.pdf]

ADVANCED MATERIALS

Supporting Information

for *Adv. Mater.*, DOI 10.1002/adma.202411780

Integration of Through-Sapphire Substrate Machining with Superconducting Quantum Processors

*Narendra Acharya, Robert Armstrong, Yashwanth Balaji, Kevin G. Crawford, James C. Gates, Paul C. Gow, Oscar W. Kennedy, Renuka Devi Pothuraju, Kowsar Shahbazi and Connor D. Shelly**

Supplemental Information for: Integration of Through-Sapphire Substrate Machining with Superconducting Quantum Processors

*Narendra Acharya Robert F. Armstrong Yashwanth Balaji Kevin G. Crawford James C. Gates Paul C. Gow Oscar W. Kennedy Renuka Devi Pothuraju Kowsar Shahbazi Connor D. Shelly**

N. Acharya, R. F. Armstrong, Y. Balaji, K. G. Crawford, O. W. Kennedy, R. D. Pothuraju, K. Shahbazi, C. D. Shelly
Oxford Quantum Circuits, Thames Valley Science Park, Shinfield, Reading, United Kingdom, RG2 9LH
Email Address: cshelly@oqc.tech
J. C. Gates, P. C. Gow
Optoelectronics Research Centre, University of Southampton, Southampton SO17 1BJ, UK

1 Full-wafer JJ Spread

Through recent fabrication improvements our 3" wafer-scale spread as fabricated, inclusive of sapphire machining and dicing, is as low as 2.4% - in-line with the best reported as-fabricated values in the literature (for instance see [1]). Note that qubits from this wafer have not been cryogenically tested so no coherence values are reported. The total yield of this wafer was approximately 80% due to ESD failures. In this wafer we also note that we see less resistance outliers than in the wafer shown in the main manuscript. We do sometimes see resistance outliers that could be biased to higher or lower resistance. The mechanism for this is not completely clear, nor necessarily the same for each occurrence, but is very likely due to common fabrication variations in wafer scale processing. Such variations may include (but are not limited to), surface contamination, lithography defects, resist residue, and deposition variations.

2 Demonstration of through-sapphire-vias

We also demonstrate the metalisation of the through-sapphire-apertures to produce proof-of-principle through-sapphire-vias. The apertures are metalised from both sides using an electron-beam deposition tool to deposit approximately 300 nm of aluminium through the apertures. An argon milling step is included between the two depositions to ensure ohmic contact, as vacuum is broken in this process. The substrate is rotated throughout the deposition to ensure full aperture wall coverage. Two-probe room temperature resistance measurements between the top and bottom ground planes show a resistance of $7\,\Omega$ demonstrating conduction through the via. Alternative methods of metalising may include means such as sputtering, or by cold-metal injection [2].

3 Laser Drilling Integration

It is possible to drill apertures in sapphire using a laser drilling process. As this technique is ablative, the substrate will get hot during the drilling. This thermal effect can be a limitation for substrates with devices that are affected by heat (for quantum circuits this is important as heat can change the parameters of the Josephson junction). In addition, if any resist is used for protection or further processing then this can lead to thermal cross-linking of the resist causing difficulty in removal. In order to integrate this to a process flow in which the JJs are already manufactured protective resist is required. Figure S3 shows that the laser drilling results in thermal cross-linking of both PMMA and S1813 resists. Attempts at removal using Acetone, NMP, and finally O2 plasma etching were unsuccessful. As resist residue is detrimental to qubit coherence this is not a favourable process for the integration of through-sapphire apertures in quantum circuits [3]. In addition, the large-distance heating will result in JJ resistance changes and thus further difficulty with precise frequency allocations in QPUs.

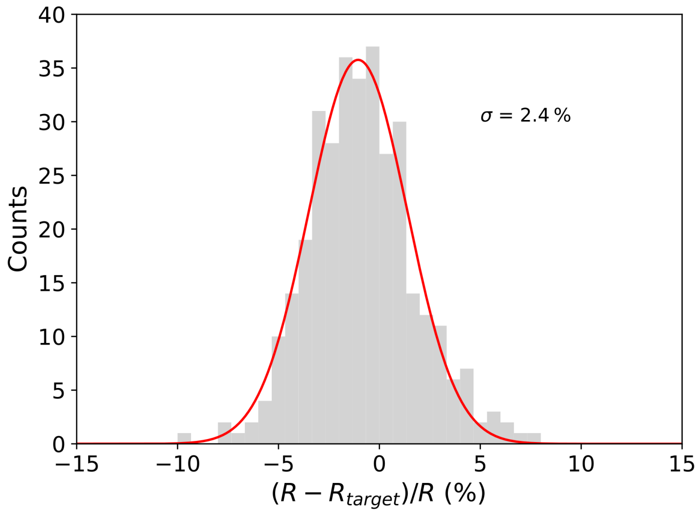

Figure S1: Histogram of Josephson junction resistances measured on a 3" wafer of Toshiko 32Q QPUs. The histogram is a composite of three sets of JJs normalised to their median, or target value R_{target}. The composite spread in resistance is 2.4 %

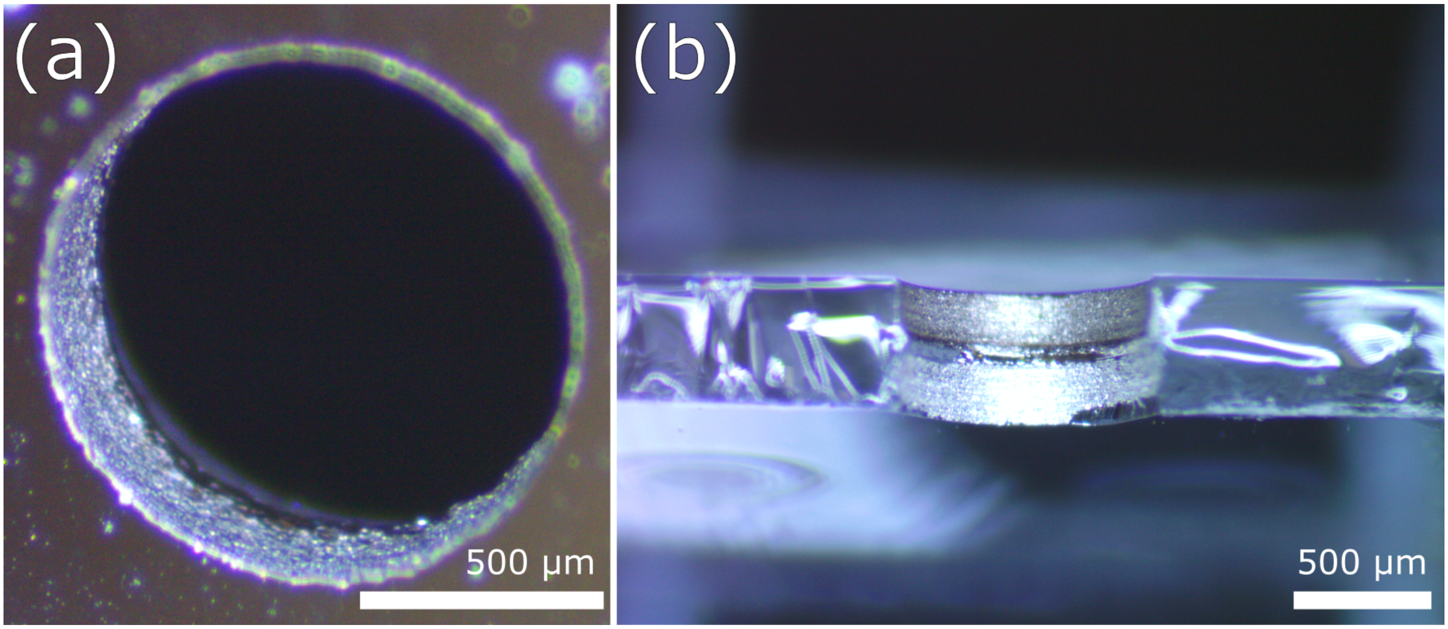

Figure S2: Micrographs of machined and metal-coated via. (a) Micrograph of 1 mm diameter drilled aperture with aluminium deposited via electron-beam deposition onto the aperture sidewall. (b) Micrograph of a cleaved sapphire substrate showing the sidewalls of the 1 mm diameter drilled apertures post aluminium metal deposition. A two-probe resistance measurement showed a 7 Ω resistance through the via.

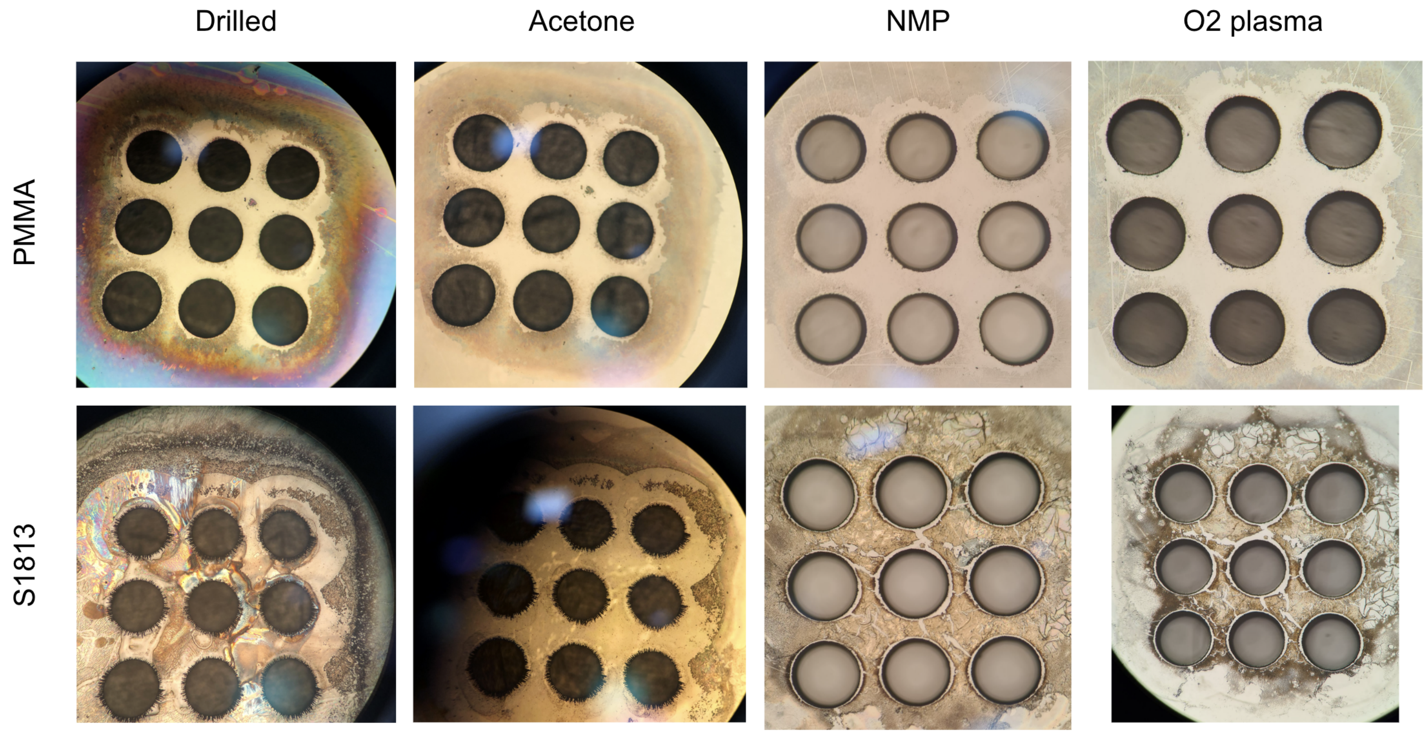

Figure S3: Micrographs of laser-drilled 1 mm apertures and attempts at resist removal. (a) Optical micrograph of electron-beam resist (top row) and photoresist (bottom row) after laser drilling a 3x3 matrix of apertures in the sapphire substrate. The figures show the resist residue remaining after various cleans - acetone (column 2), NMP (column 3) and O2 plasma (column 4). In each case residue remains and cannot be completely removed.

4 Resistance Change vs Distance from Aperture

We show the radial trend in resistance change across the drilling process. Any trend is substantially smaller than the junction-to-junction variability present in our data meaning any radial variation, if present, is weak. All qubits in our Toshiko lattice are 2 mm from the nearest hole, i.e. off the panel in this graph, giving us good confidence that drilling will make undetectable changes to the properties of the Josephson junctions in our qubits.

5 Temporal Fluctuations of $T1$

We present an example data set of the $T1$ coherence time fluctuating over a time period of approximately 18 hours. This is a representative data set from one of the qubits presented in Fig 3b in the main manuscript.

References

[1] A. Osman, J. Fernández-Pendás, C. Warren, S. Kosen, M. Scigliuzzo, A. Frisk Kockum, G. Tancredi, A. Fadavi Roudsari, J. Bylander, *Phys. Rev. Res.* **2023**, *5* 043001.

[2] S. S. Ahmad, F. Haring, A. Reinholz, N. Schneck, *IMAPSource Proceedings* **2014**, *2014*, DPC 1422.

[3] J. Lisenfeld, A. Bilmes, A. Megrant, R. Barends, J. Kelly, P. Klimov, G. Weiss, J. M. Martinis, A. V. Ustinov, *npj Quantum Information* **2019**, *5*, 1 105.

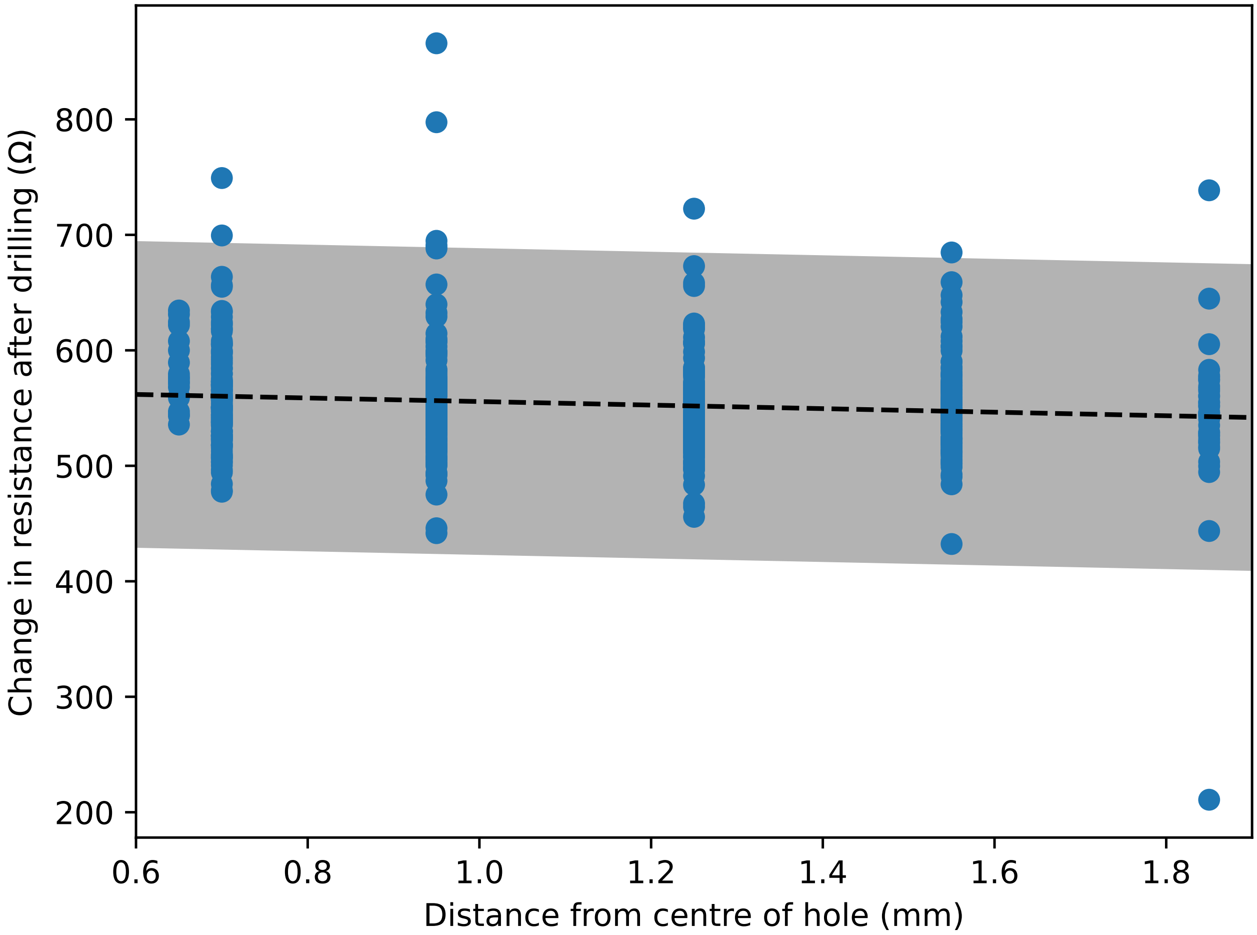

Figure S4: Change in resistance after the drilling process is plotted as a function of distance between the junction and the centre of the hole for data presented in Fig. 2. We fit a line to this data and shade +/- one standard deviation of the as-fabricated resistance above and below the line.

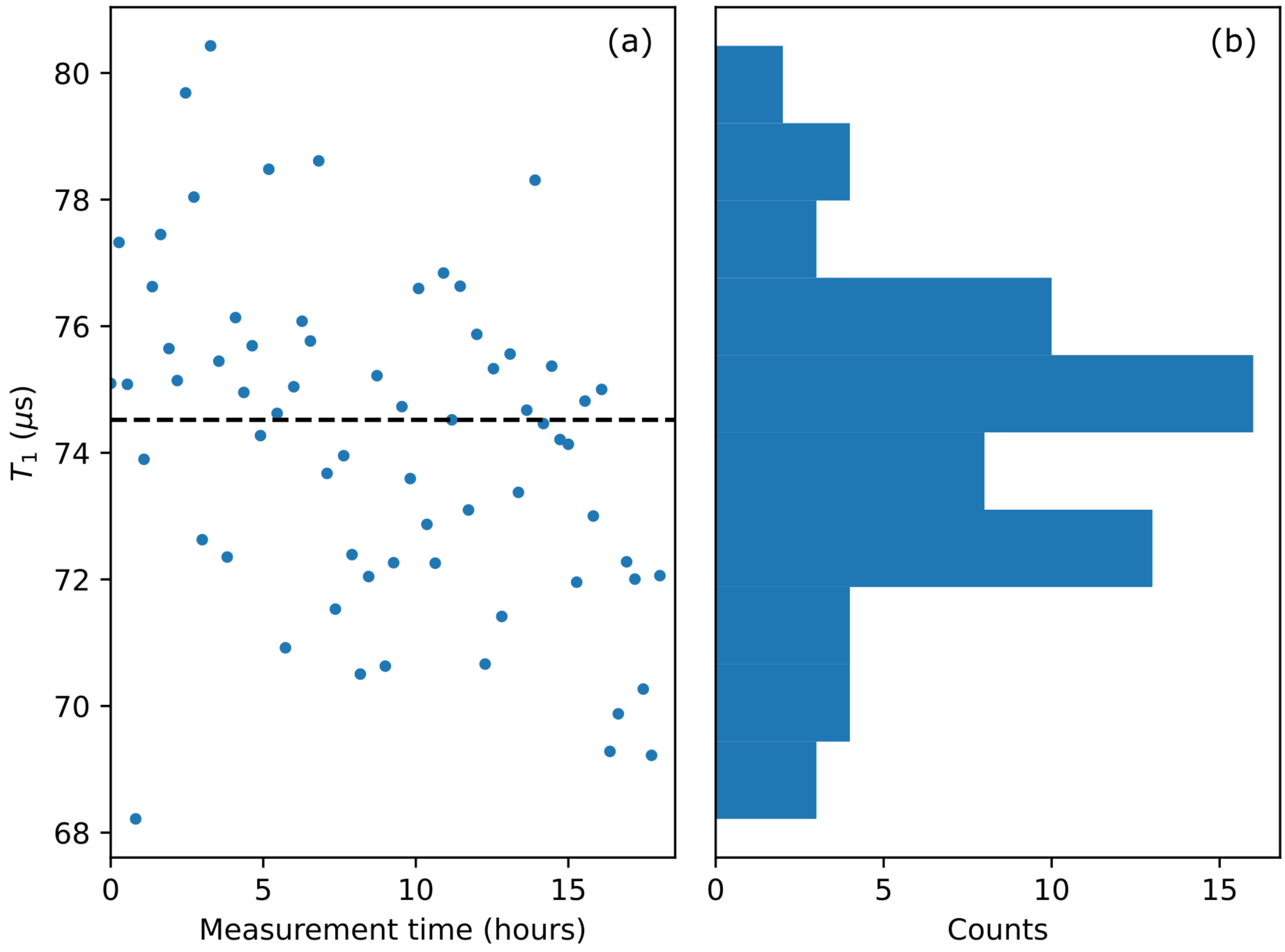

Figure S5: (a) Temporal fluctuations in $T1$ over a time period of approximately 18 hours. (b) A histogram of the $T1$ data.
